# Multi-Omics and Functional Validation Identify a Quercetin-SLC15A2 Axis That Mediates the Anti-Fibrotic Effect of Shen-Kang Recipe in Diabetic Kidney Disease

**DOI:** 10.3390/ijms27073291

**Published:** 2026-04-05

**Authors:** Anna Zuo, Shuyu Li, Jiarun Xie, Lishan Huang, Ziwei Li, Jingxin Lin, Xiaoshan Zhao, Ming Wang

**Affiliations:** 1 School of Traditional Chinese Medicine, Southern Medical University, Guangzhou 510515, China; zuo199720@163.com (A.Z.); 18312171688@163.com (J.X.); 18529237359@163.com (Z.L.); 3178010069@smu.edu.cn (J.L.); 2 Artemisinin Research Center, Institute of Chinese Materia Medica, China Academy of Chinese Medical Sciences, Beijing 100700, China; 18637836720@163.com; 3 School of Pharmacy, Jinan University, Guangzhou 510632, China; huanglishan302@163.com

**Keywords:** SLC15A2, quercetin, renal fibrosis, diabetic kidney disease, Shen-Kang Recipe, multi-omics, quantitative proteomics, molecular dynamics simulations

## Abstract

Diabetic kidney disease (DKD) is a leading cause of end-stage renal disease. The Shen-Kang Recipe (SKR) is a traditional Chinese medicine formula used clinically to slow DKD progression, but its bioactive constituents and molecular targets remain unclear. Solute carrier family 15 member 2 (SLC15A2/PEPT2), a high-affinity peptide transporter expressed in renal proximal tubules, has been implicated in kidney pathophysiology, yet its potential role in mediating the therapeutic effects of the SKR has not been explored. Here, we evaluated the effects of the SKR in db/db mice and found that SKR treatment significantly improved renal function, attenuated glomerulosclerosis, and reduced interstitial collagen deposition. Wide-target metabolomics and quantitative proteomics revealed that the SKR broadly reversed DKD-associated metabolic and proteomic disturbances, particularly in pathways related to energy and amino acid metabolism. Proteomic analysis identified SLC15A2 as a key proximal tubule protein downregulated in DKD and selectively restored by the SKR. UPLC-Q-TOF/MS-based serum pharmacochemistry and network pharmacology highlighted quercetin as a principal bioactive component of the SKR. Molecular docking, molecular dynamics simulations, and surface plasmon resonance (SPR) confirmed direct, high-affinity binding between quercetin and SLC15A2 (KD = 7.5 µM). In TGF-β1-stimulated HK-2 cells, quercetin suppressed epithelial-mesenchymal transition (EMT), as evidenced by restored E-cadherin and reduced N-cadherin, vimentin, and α-SMA expression; this effect was abrogated by siRNA-mediated SLC15A2 knockdown, demonstrating the functional necessity of this axis. Collectively, these findings identify a quercetin-SLC15A2 axis through which the SKR inhibits EMT and alleviates renal fibrosis in DKD, providing a mechanistic basis for its clinical application and nominating SLC15A2 as a potential therapeutic target.

## 1. Introduction

Diabetic kidney disease (DKD) is a common and debilitating microvascular complication of diabetes and remains the leading cause of end-stage renal disease (ESRD) worldwide [1,2]. DKD is characterized by glomerular hypertrophy, basement membrane thickening, mesangial expansion, and progressive tubulointerstitial fibrosis, ultimately leading to irreversible loss of kidney function [3]. Although intensive glycemic and blood pressure control and renin–angiotensin–aldosterone system blockade can slow progression, many patients continue to develop advanced chronic kidney disease [4,5]. Therefore, elucidating DKD pathogenesis and identifying actionable therapeutic targets remain urgent.

Traditional Chinese medicine (TCM) offers a holistic, multi-target approach supported by extensive clinical experience [6,7,8]. The Shen-Kang Recipe (SKR) is an empirical TCM formula composed of eleven medicinal herbs that has shown promising potential in delaying DKD progression [9]. Quercetin, an abundant flavonoid in the SKR, exhibits antioxidant, anti-inflammatory, and anti-fibrotic activities [10,11] and has been reported to reduce extracellular matrix deposition and renal fibrosis through pathways including Nrf2 and NF-kB [12,13,14]. These multi-target mechanisms establish quercetin as a crucial agent within the SKR for countering the multifaceted pathology of DKD [15]. Nevertheless, the key molecular targets through which the SKR and its constituents confer renoprotection remain to be defined.

Recent advances in systems biology and computational pharmacology enable integrative strategies to decipher the mechanisms of multi-component herbal formulations [16,17]. In this study, we combined widely targeted metabolomics, 4D label-free quantitative proteomics, serum pharmacochemistry, network pharmacology, and computational simulations to systematically dissect the mode of action of the SKR [18,19,20].

Solute carrier family 15 member 2 (SLC15A2), commonly known as peptide transporter 2 (PEPT2), is a high-affinity peptide transporter predominantly localized to renal proximal tubules, where it mediates the reabsorption of di- and tripeptides as well as various peptidomimetic drugs [21,22]. Emerging evidence implicates SLC15A2 in diverse physiological and pathological processes, including neuroprotection [23,24] and the handling of uremic toxins in chronic kidney disease [25,26]. Hyperglycemia and metabolic disturbances may regulate its expression [27], and it may exert anti-inflammatory and anti-fibrotic effects via clearance of pathogenic peptides [28,29]. However, whether SLC15A2 participates in DKD progression and can be therapeutically leveraged by herbal interventions remains unclear.

In this study, we employed db/db mice and HK-2 cell models to show that the SKR improves renal function and attenuates fibrosis and identify the quercetin-SLC15A2 axis as a central pathway underpinning these benefits.

## 2. Results

### 2.1. SKR Ameliorates Renal Function in Db/Db Mice

**Figure 1:**
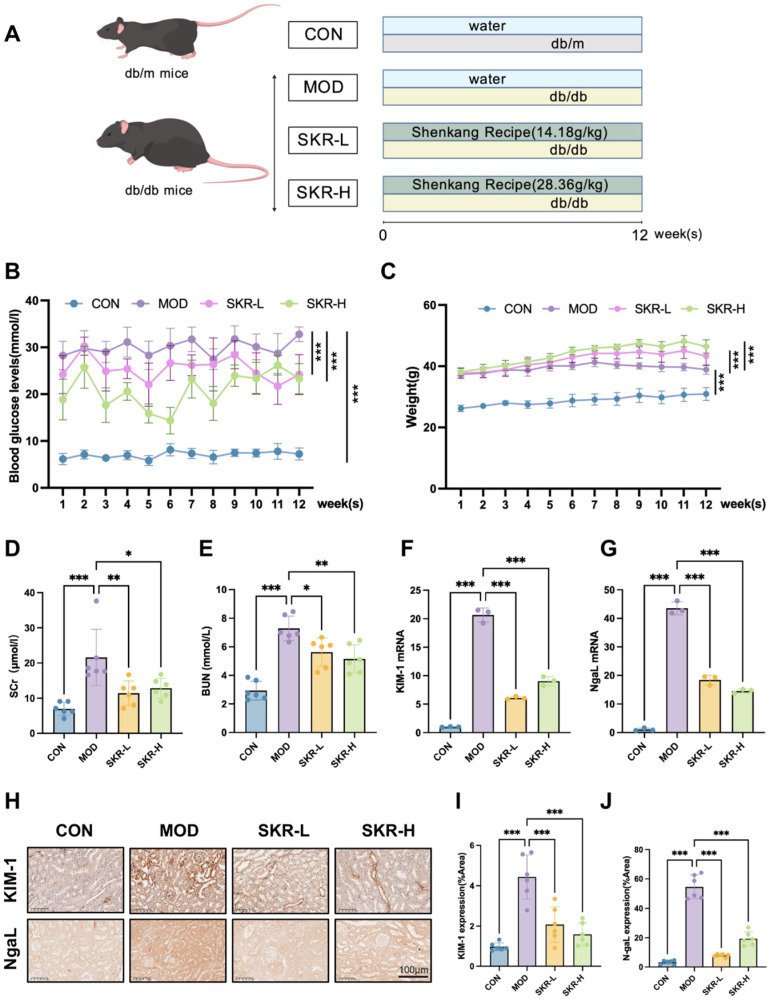
The SKR ameliorates hyperglycemia and renal function in db/db mice. (**A**) Schematic diagram of animal experimental design. MOD refers to the model group (db/db mice), while SKR-L and SKR-H represent the low-dose and high-dose Shen-Kang Recipe treatment groups. (**B**) Changes in blood glucose levels in mice treated with different medicine. Each dot represents one data point. (**C**) Changes in body weight in mice treated with different medicine. (**D**) Serum Cr levels. (**E**) Serum BUN levels. (**F**,**G**) RT-qPCR analysis of renal *KIM-1* and *NgaL* mRNA levels. (**H**) IHC detection of renal KIM-1 and NgaL expression. Scale bar = 200 μm. (**I**) Statistical analysis of KIM-1 expression. (**J**) Statistical analysis of NgaL expression. All data were presented as mean ± SD, *n* = 6. * *p* < 0.05, ** *p* < 0.01, and *** *p* < 0.001.

To evaluate the therapeutic potential of the SKR in DKD, db/db mice were divided into three groups: model (MOD), low-dose SKR (SKR-L, 14.18 g/kg/day), and high-dose SKR (SKR-H, 28.36 g/kg/day). These mice were treated via gavage for 12 weeks, with db/m mice serving as normal controls and saline-treated db/db mice as model controls (Figure 1A). Detailed experimental procedures are described in Section 4.1. Compared with the model group, the SKR reduced random blood glucose levels (Figure 1B) and partially improved body weight gain (Figure 1C). The SKR also lowered serum creatinine and blood urea nitrogen levels (Figure 1D,E). Consistently, the SKR decreased renal KIM-1 and NGAL mRNA expression (Figure 1F,G) and reduced their protein expression by immunohistochemistry (Figure 1H–J). Together, these data indicate that the SKR improves metabolic status and confers renoprotection in db/db mice.

### 2.2. SKR Reduces Renal Inflammation and Fibrosis in Db/Db Mice

**Figure 2:**
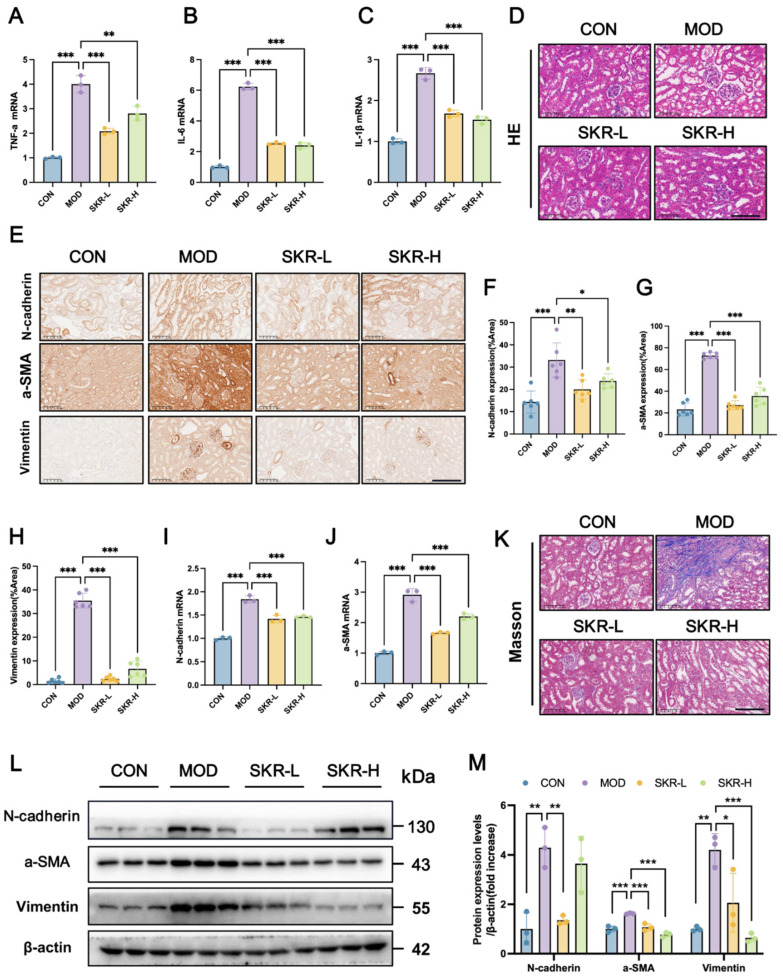
SKR ameliorates renal inflammation and fibrosis in db/db mice. (**A**–**C**) Renal *TNF-α*, *IL-6*, and *IL-1β* mRNA levels. (**D**) H&E staining of renal tissue. Scale bar = 100 μm. (**E**) IHC detection of renal N-cadherin, α-SMA, and Vimentin expression. Scale bar = 100 μm. (**F**–**H**) Statistical analysis of N-cadherin, α-SMA, and Vimentin expression from IHC. (**I**,**J**) RT-qPCR analysis of renal *N-cadherin* and *α-SMA* mRNA levels. (**K**) Masson staining of renal tissue. Scale bar = 100 μm. (**L**) WB detection of renal N-cadherin, α-SMA, and vimentin protein levels. (**M**) Statistical analysis of N-cadherin, α-SMA, and vimentin protein expression. All data were presented as mean ± SD, *n* = 3. Each dot represents one data point. * *p* < 0.05, ** *p* < 0.01, and *** *p* < 0.001.

To elucidate the renoprotective mechanisms of the SKR, we investigated its effects on inflammatory and fibrotic pathways in renal tissues. SKR treatment significantly downregulated mRNA expression of pro-inflammatory cytokines, including *IL-6*, *IL-1β* and *TNF-α* (Figure 2A–C). Histological analysis via H&E staining confirmed substantial reduction in inflammatory cell infiltration and architectural damage (Figure 2D). Concurrently, the SKR demonstrated significant anti-fibrotic efficacy. Renal tissues from db/db model mice exhibited substantial upregulation of the myofibroblast marker α-SMA and interstitial fibrosis markers (N-cadherin and vimentin), as confirmed at both transcriptional and protein levels by immunohistochemical (Figure 2E–H), qPCR (Figure 2I,J) and Western blot analyses (Figure 2L,M). This molecular evidence of fibrotic activation was further corroborated by Masson’s trichrome staining, which revealed extensive collagen deposition in the model group (Figure 2K). Notably, SKR treatment significantly attenuated these fibrotic alterations, effectively suppressing the expression of key fibrosis indicators and reducing collagen accumulation in renal tissues. These findings collectively demonstrate that the SKR effectively delays renal fibrosis progression in db/db mice by inhibiting myofibroblast activation and interstitial phenotype expression, suggesting that its mechanism may involve the regulation of epithelial-mesenchymal transition (EMT) processes.

### 2.3. Proteomics Identification of Key Targets Mediating SKR’s Improvement in DKD

**Figure 3:**
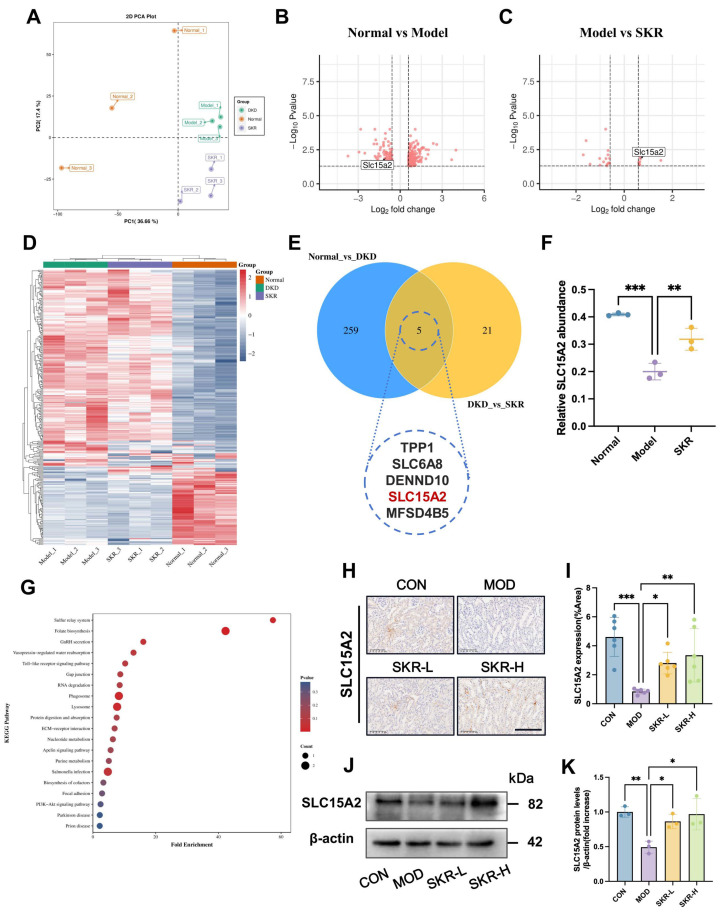
Proteomic identification of key targets of the SKR in the treatment of diabetic kidney disease. (**A**) Principal component analysis (PCA) of renal proteomes from the control (orange), model (green), and SKR-treated (purple) groups; PC1 and PC2 represent 36.66% and 17.4% of the total variance, respectively. (**B**) Volcano plot of differentially expressed proteins (DEPs) between the control and model groups. Red dots represent significantly upregulated and downregulated proteins, respectively. (**C**) Volcano plot of DEPs between the model and SKR-treated groups. Red dots indicate significantly upregulated or downregulated proteins (adjusted *p*-value < 0.05, |log_2_FC| > 0.585). SLC15A2 is highlighted in the plot. Each dot represents an individual gene. (**D**) Hierarchical clustering heatmap of all identified DEPs across groups. Significance thresholds are the same as in (**B**). (**E**) Venn diagram showing the overlap of differentially expressed proteins identified from the Normal vs. DKD and DKD vs. SKR comparisons. Five overlapping proteins are highlighted as potential key targets. (**F**) Relative expression level of SLC15A2 in renal tissues based on proteomic data. (**G**) Top 20 enriched KEGG pathways among the identified DEPs. (**H**) Representative immunohistochemistry images of SLC15A2 in kidney sections. Scale bar = 100 μm. (**I**) Quantification of SLC15A2 expression from IHC. (**J**) Western blot analysis of SLC15A2 protein levels. (**K**) Densitometric quantification of SLC15A2 from Western blot. Data are presented as mean ± SD, *n* = 3. * *p* < 0.05, ** *p* < 0.01, and *** *p* < 0.001.

To elucidate the novel mechanisms underlying the renoprotective effects of the SKR in DKD, we conducted 4D label-free quantitative proteomic analysis on renal tissues from normal control (NC), db/db model (Model), and high-dose SKR-treated (SKR) groups. Principal Component Analysis (PCA) revealed distinct separation among the three sample groups, indicating significant differences in their protein expression profiles (Figure 3A). Volcano plot analysis revealed a substantial number of differentially expressed proteins (DEPs) in the Model group compared to the NC group. SKR intervention significantly modulated the expression of a subset of these DEPs (Figure 3B,C). Through comparative screening, we identified five candidate proteins potentially regulated by the SKR (Figure 3E), among which tripeptidyl peptidase 1 (TPP1), creatine transporter 1 (SLC6A8), and solute carrier family 15 member 2 (SLC15A2) have established associations with renal pathophysiology [30,31,32]. Notably, SLC15A2, a proton-coupled oligopeptide transporter primarily expressed in renal proximal tubules, is responsible for the reabsorption of dipeptides and tripeptides, as well as the uptake of certain uremic toxins [33]. Heatmap visualization and quantitative analysis demonstrated that SLC15A2 expression was significantly downregulated in the Model group compared to the NC group, and was effectively restored to near-normal levels following high-dose SKR treatment (Figure 3D,F). Specifically, SLC15A2 was significantly downregulated in the Model group compared to the Normal group (log_2_FC = −1.03, *p* = 0.0132), and was significantly upregulated following SKR treatment compared to the Model group (log_2_FC = 0.67, *p* = 0.0147). KEGG pathway enrichment analysis (Figure 3G) indicated that these DEPs were significantly enriched in pathways such as ECM-receptor interaction, Focal adhesion, Protein digestion and absorption, and the PI3K-Akt signaling pathway. These pathways are known to play critical roles in diabetic kidney disease progression, including extracellular matrix accumulation, tubular injury, and inflammatory responses [34]. Given that SLC15A2 is a key mediator of protein reabsorption and toxin accumulation in proximal tubules, its enrichment in these pathways provides a mechanistic context for its potential role in DKD.

To validate the proteomic findings, we examined SLC15A2 protein expression using Western blot and immunohistochemistry. The results closely matched the proteomic data: SLC15A2 expression was markedly reduced in db/db mice, and SKR treatment effectively restored its expression (Figure 3H–K). In summary, our integrated proteomic and biochemical analyses demonstrate that SLC15A2 downregulation is associated with DKD pathogenesis, and that the SKR exerts its renoprotective effect at least partially through upregulation of SLC15A2.

### 2.4. Wide-Target Renal Metabolomics Reveals the Metabolic Remodeling Effect of SKR in Ameliorating DKD

**Figure 4:**
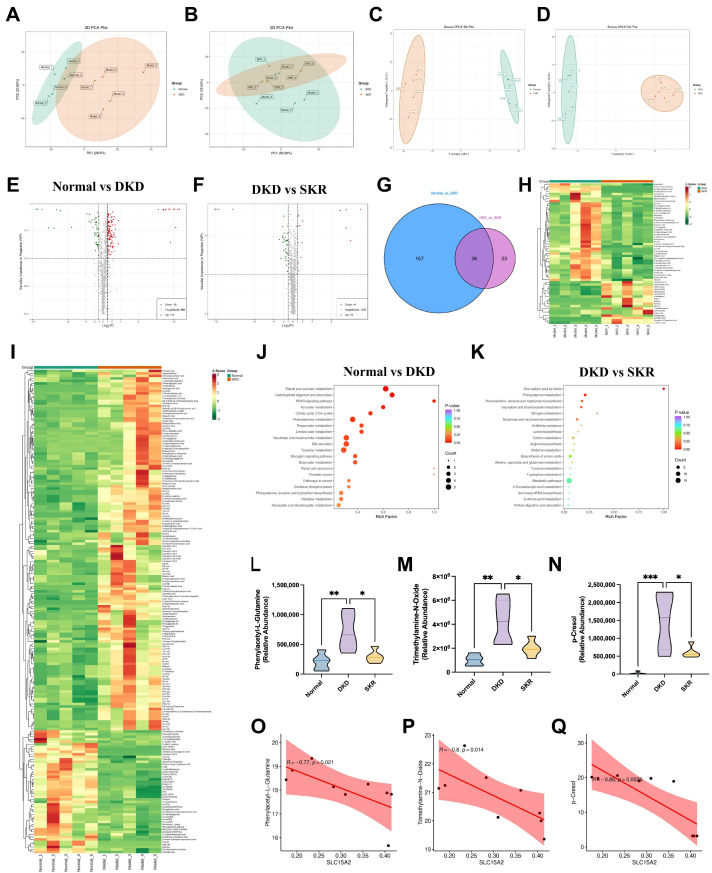
Wide-targeted metabolomics identifies key metabolites underlying the therapeutic effect of the SKR on diabetic kidney disease. (**A**,**B**) PCA score plots showing the separation among the control, model, and SKR-treated groups and the reversal trend induced by SKF treatment in renal metabolic profiles. (**C**,**D**) OPLS-DA score plots demonstrating the discrimination among groups and the metabolic restoration by SKR treatment. (**E**,**F**) Volcano plots for screening differential metabolites. Significantly down-regulated and up-regulated metabolites are shown in green and red, respectively; metabolites detected without significant changes are shown in gray. The x-axis represents the log_2_ fold change (log_2_FC) between groups. (**G**) Venn diagram of differential metabolites. (**H**,**I**) Hierarchical clustering heatmaps of differential metabolites in kidney tissues across groups. (**J**,**K**) KEGG enrichment analysis of differential metabolites. (**L**–**N**) Changes in kidney levels of key gut-kidney axis toxins: N-phenylacetyl-L-glutamine (PAGln), trimethylamine N-oxide (TMAO), and p-cresol. The central line of each box indicates the median. (**O**–**Q**) Correlation analysis between key differential metabolites and expression of the core target protein SLC15A2. The red line indicates the linear regression fit. Data are presented as mean ± SD, *n* = 5. * *p* < 0.05, ** *p* < 0.01, and *** *p* < 0.001.

To investigate the impact of the SKR on renal endogenous metabolites, we performed metabolomics analysis. Both principal component analysis (PCA) and partial least squares-discriminant analysis (PLS-DA) models demonstrated a distinct separation in metabolic profiles between the DKD model group and the control group. The SKR treatment group exhibited a clear trend of reversion towards the normal group, indicating that the SKR significantly ameliorates the systemic metabolic disturbances induced by DKD (Figure 4A–D). We identified a total of 36 metabolites with significant differences among the groups (VIP > 1, *p* < 0.05), encompassing various gut-kidney axis toxins, amino acid metabolites, and peptide substrates, among others (Figure 4E–G). Heatmap analysis provided a visual representation of the alterations in these metabolites before and after SKR intervention (Figure 4H,I). Pathway enrichment analysis indicated that these differential metabolites were primarily involved in key metabolic pathways, including Phenylalanine metabolism, Tyrosine metabolism, Tryptophan metabolism, Histidine metabolism, Alanine, aspartate and glutamate metabolism, and Protein digestion and absorption (Figure 4K). Perturbations in pathways related to energy metabolism and amino acid homeostasis, notably involving metabolites such as Phenylacetyl-L-Glutamine (PAGln), Trimethylamine-N-Oxide (TMAO), and p-Cresol, were significantly corrected following SKR treatment [35,36,37] (Figure 4L–N). Further analysis revealed a negative correlation between the expression levels of the key proximal tubule peptide transporter SLC15A2 and the circulating levels of PAGln, TMAO, and p-Cresol (Figure 4O–Q). Collectively, these metabolomic findings demonstrate that the SKR reverses DKD-associated metabolic disturbances, particularly in uremic toxin-related pathways, and that this effect may be linked to modulation of SLC15A2 expression. This action could contribute to restoring renal energy metabolism and amino acid utilization, thereby improving cellular function and inhibiting EMT and fibrosis.

### 2.5. Identification of Key Constituents of SKR for DKD via an Integrated Multi-Technique Approach

**Figure 5:**
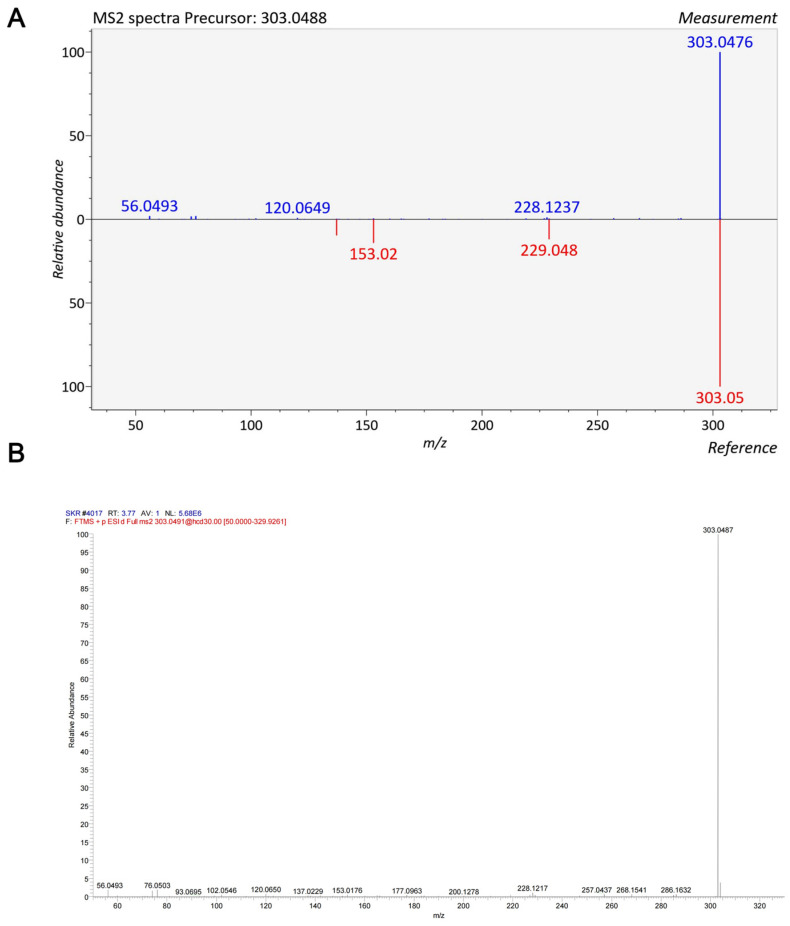
Mass spectrometric identification of quercetin in SKR-containing serum. (**A**) Mirror plot of full-scan mass spectra (positive ion mode) of quercetin standard (upper panel) and serum sample from SKR-treated mice (lower panel). The protonated molecular ion [M + H]^+^ at *m*/*z* 303.05 is observed in both, with identical isotopic distribution, confirming the presence of quercetin in systemic circulation. (**B**) Mirror plot of MS/MS spectra (positive ion mode) of quercetin standard (upper) and the same serum sample (lower). Characteristic fragment ions at *m*/*z* 285.04 ([M + H–H_2_O]^+^), 257.04 ([M + H–H_2_O–CO]^+^), and 229.05 are identical between the standard and the sample, unequivocally identifying quercetin as a prototype component absorbed after oral administration of the SKR.

To elucidate the pharmacologically active constituents of the SKR, we employed an integrated multi-technique strategy. First, network pharmacology analysis was performed to construct a compound-target interaction network for the SKR (Figure S1). Topological analysis based on degree centrality identified quercetin as the most highly connected node (a complete list of key components is provided in Supplementary Table S1). The targets predicted for quercetin were significantly enriched in pathways associated with renal metabolism and inflammation, highlighting its potential central role from a computational standpoint [38,39]. Next, we systematically characterized the chemical profile of the SKR aqueous extract using ultra-high-performance liquid chromatography-quadrupole time-of-flight mass spectrometry (UPLC-Q-TOF/MS). The total ion chromatogram of the extract is provided in Supplementary Figure S2, confirming quercetin as one of the major constituents. To verify its in vivo bioavailability, we analyzed serum samples from SKR-treated mice. As shown in Figure 5A, the protonated molecular ion [M + H]^+^ of quercetin at *m*/*z* 303.05 was detected in both the quercetin standard and the SKR-containing serum sample, with identical isotopic distribution, confirming the presence of quercetin in the systemic circulation. To further verify its structural identity, MS/MS fragmentation was performed. The representative MS/MS spectrum (Figure 5B) displayed characteristic fragment ions of quercetin at *m*/*z* 285.04 ([M + H–H_2_O]^+^), 257.04 ([M + H–H_2_O–CO]^+^), and 229.05, which matched perfectly with the fragmentation pattern of the quercetin standard. These data unequivocally demonstrate that quercetin is a prototype component absorbed into systemic circulation after oral administration of the SKR, providing a pharmacokinetic basis for its potential direct action on target organs such as the kidney. Thus, quercetin was identified as a key bioactive constituent of the SKR that is absorbed into systemic circulation and likely contributes to the therapeutic effects against DKD.

### 2.6. Quercetin Reverses TGF-β1-Induced EMT in HK-2 Cells In Vitro

**Figure 6:**
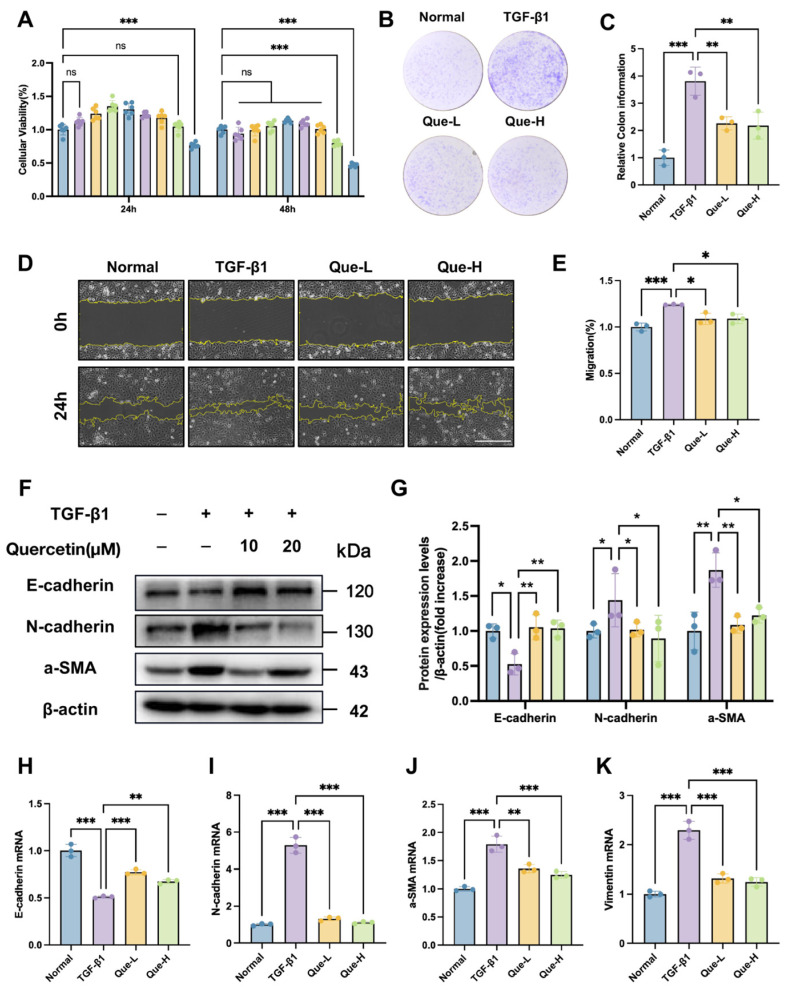
The active component quercetin in the SKR inhibits EMT in HK-2 cells. (**A**) Viability of HK-2 cells treated with various concentrations of quercetin for 24 and 48 h. Each dot = one replicate well. Concentrations (1.25–300 μM from left to right) are color-coded as shown in the legend. Colors may repeat across concentrations. (**B**) Representative images of colony formation assay. (**C**) Quantitative analysis of colony formation. (**D**) Representative images of wound healing assay at 0 and 24 h. Yellow dashed lines indicate wound edges. Scale bar = 100 μm. (**E**) Quantitative analysis of wound closure rate at 24 h. (**F**) Western blot analysis of E-cadherin, N-cadherin and α-SMA expression. (**G**) Quantitative analysis of E-cadherin, N-cadherin and α-SMA protein levels. (**H**–**K**) RT-qPCR analysis of *E-cadherin*, *N-cadherin*, *α-SMA* and *Vimentin* mRNA levels in HK-2 cells. Data are presented as mean ± SD, *n* = 3. * *p* < 0.05, ** *p* < 0.01, and *** *p* < 0.001, ns indicates not significant, *p* ≥ 0.05.

**Figure 7:**
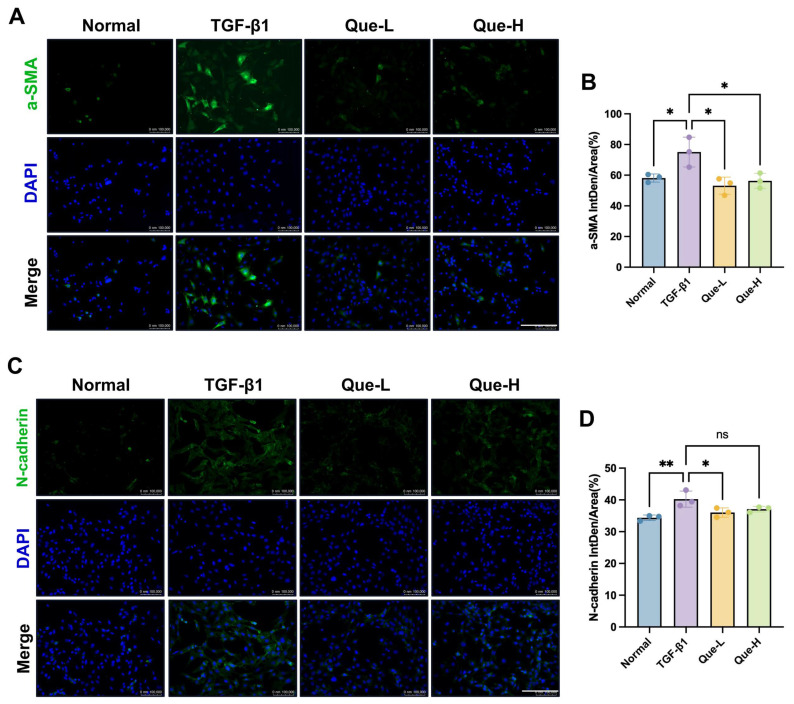
Quercetin inhibits TGF-β1-induced epithelial-mesenchymal transition in HK-2 cells. (**A**) Representative immunofluorescence images of α-SMA (green) in each group. Nuclei were counterstained with DAPI (blue). Scale bar = 100 μm. (**B**) Quantitative analysis of α-SMA fluorescence signal. (**C**) Representative immunofluorescence images of N-cadherin (green) in each group. Nuclei were counterstained with DAPI (blue). Scale bar = 100 μm. (**D**) Quantitative analysis of N-cadherin fluorescence signal. All data were presented as mean ± SEM, *n* = 3. Each dot represents one replicate. * *p* < 0.05, ** *p* < 0.01, ns indicates not significant, *p* ≥ 0.05.

Following the identification of quercetin as the key SKR component interacting with SLC15A2, we further investigated its anti-fibrotic efficacy in a TGF-β1-induced HK-2 cell model. The CCK-8 assay confirmed that quercetin exhibited no significant cytotoxicity at concentrations up to 80 μM (Figure 6A). Based on these results, doses of 10 and 20 μM were selected for subsequent experiments. Functional assessments demonstrated that quercetin also significantly suppressed TGF-β1-induced cell proliferation (colony formation assay) and migration (wound healing assay) in HK-2 cells (Figure 6B–E). To elucidate the underlying molecular mechanism, we examined fibrosis-related markers via qPCR and Western Blot. The results indicate that quercetin significantly reversed TGF-β1-induced alterations in key protein expression, notably upregulating the epithelial marker *E-cadherin* while downregulating the mesenchymal markers *N-cadherin*, *Vimentin* and *α-SMA* (Figure 6F–K). Furthermore, immunofluorescence staining provided additional confirmation at the protein localization level, showing the inhibitory effect of quercetin on the expression of α-SMA and N-cadherin (Figure 7A–D). Taken together, these results demonstrate that quercetin, as a core bioactive component of the SKR, attenuates TGF-β1-induced fibrotic changes in HK-2 cells by suppressing the epithelial-mesenchymal transition process.

### 2.7. Quercetin Directly Targets and Stably Binds to the Protective Transporter SLC15A2

**Figure 8:**
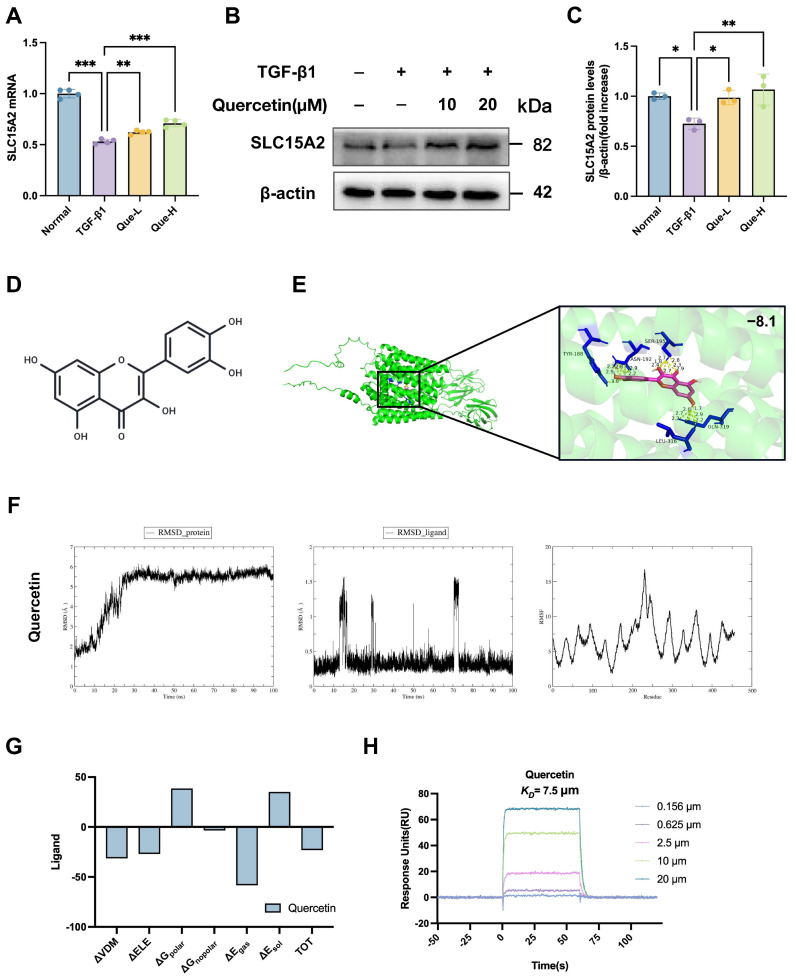
Quercetin directly targets and stably binds to the protective transporter SLC15A2. (**A**) *SLC15A2* mRNA expression analyzed by RT-qPCR. (**B**) Western blot analysis of SLC15A2 protein expression. (**C**) Quantitative analysis of SLC15A2 protein levels. (**D**) The molecular structural formula of quercetin. (**E**) Molecular docking reveals the binding mode of quercetin to SLC15A2. (**F**) Computed root mean square deviation and root mean square fluctuation values of Cα atoms in each system for quercetin with SLC15A2. (**G**) Binding free energies of SLC15A2 complexes with the aforementioned molecules. (**H**) SPR analysis confirms the high-affinity direct binding between quercetin and SLC15A2 protein. Real-time sensorgrams (Response Units, RU) showing the binding of increasing concentrations of quercetin (from bottom to top) to the immobilized SLC15A2 protein. Equilibrium dissociation constant (KD) is 7.5 μm. All data were presented as mean ± SD, *n* = 3. Each dot represents one replicate. * *p* < 0.05, ** *p* < 0.01, and *** *p* < 0.001.

To validate whether quercetin, a key bioactive constituent of the SKR, directly interacts with the identified target SLC15A2, we conducted a series of in vitro and computational experiments. Western blot and qPCR analysis of TGF-β1-induced HK-2 cells revealed that quercetin treatment significantly upregulated the expression of SLC15A2 (Figure 8A–C). Molecular docking was performed to predict the binding mode between quercetin and SLC15A2. The results revealed that quercetin stably embeds within the substrate-binding pocket of SLC15A2, with a predicted binding energy of −8.1 kcal/mol (Figure 8E). To further assess the stability of this interaction, molecular dynamics (MD) simulations were conducted. Hydrogen bond occupancy analysis showed that the quercetin-SLC15A2 complex was primarily stabilized by a hydrogen bond between the O27 atom of quercetin and the NE1 atom of Trp405, which was present in 78.7% of the simulation trajectory. An additional hydrogen bond between the O28 atom of quercetin and the hydroxyl group of Tyr19 contributed to further stabilization, with an occupancy of 61.4% (Figure 8F; Supplementary Table S2). MM/GBSA (molecular mechanics/generalized Born surface area) calculations based on the equilibrated MD trajectory further confirmed a favorable binding free energy (Figure 8G). To quantitatively assess the binding affinity, surface plasmon resonance (SPR) analysis was performed. SPR sensorgrams showed a concentration-dependent, real-time binding of quercetin to immobilized SLC15A2 (Figure 8H). Kinetic fitting yielded an equilibrium dissociation constant (KD) of 7.5 μM, indicating a specific and high-affinity interaction. Collectively, these results demonstrate that quercetin, as a key bioactive constituent of the SKR, directly and stably binds to SLC15A2, supporting its role as a direct functional target in mediating the renoprotective effects of the SKR.

### 2.8. Loss-of-Function Validation of SLC15A2 as an Essential Mediator for the Anti-Fibrotic Effects of Quercetin

**Figure 9:**
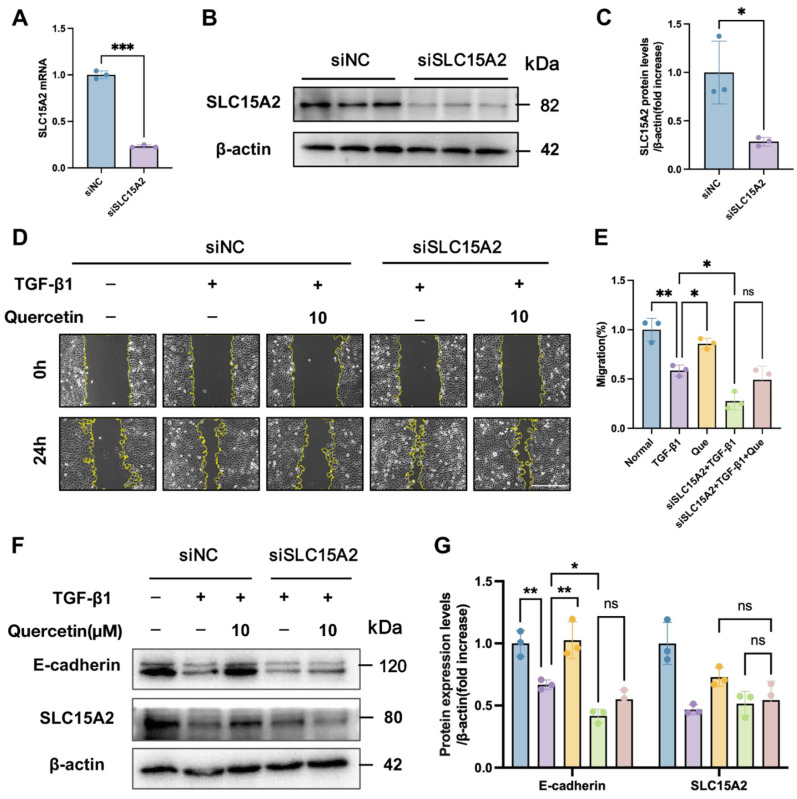
Loss-of-function validation that SLC15A2 is a necessary mediator for the anti-EMT effects of quercetin. (**A**–**C**) Efficiency validation of SLC15A2 knockdown by siRNA in HK-2 cells via qPCR and Western blot analysis. (**D**) Representative images of wound healing assay at 0 and 24 h. Yellow dashed lines indicate wound edges. Scale bar = 100 μm. (**E**) Quantitative analysis of wound closure rate at 24 h. (**F**) Western blot analysis of E-cadherin and SLC15A2 expression. (**G**) Quantitative analysis of E-cadherin and SLC15A2 protein levels. All data were presented as mean ± SD, *n* = 3. Each dot represents one replicate. * *p* < 0.05, ** *p* < 0.01, and *** *p* < 0.001, ns indicates not significant, *p* ≥ 0.05.

To establish the functional necessity of SLC15A2 in the anti-fibrotic action of quercetin, we conducted a loss-of-function experiment in HK-2 cells. Transfection with specific siRNA effectively knocked down both mRNA and protein expression of SLC15A2 (Figure 9A–C). The inhibitory effect of quercetin on TGF-β1-induced HK-2 cell migration was virtually abolished following SLC15A2 knockdown (Figure 9D,E). In the TGF-β1-induced model, quercetin treatment robustly elevated SLC15A2 protein expression in control cells, an effect that was completely abrogated in SLC15A2-deficient cells. Moreover, SLC15A2 silencing significantly impaired the regulatory capacity of quercetin over key EMT markers, essentially nullifying its ability to restore the TGF-β1-suppressed expression of E-cadherin (Figure 9F,G). These results conclusively demonstrate that SLC15A2 expression and function constitute an indispensable molecular conduit through which quercetin exerts its anti-EMT and anti-fibrotic activities.

## 3. Discussion

DKD is driven by interconnected metabolic, inflammatory, and fibrotic programs, underscoring the need for multi-target therapeutic strategies [40]. TCM formulations are well suited to this complexity because of their multi-component and multi-target characteristics, and several Chinese medicines have shown promise in DKD management [41]. The SKR is an empirically developed formula used in clinical practice and has been reported to alleviate renal senescence in DKD; however, its role and mechanism in renal fibrosis have not been fully clarified.

In the present study, we demonstrate that the SKR improves renal functional indices and markedly attenuates inflammatory infiltration and fibrotic remodeling in db/db mice, as evidenced by histology and fibrosis marker analyses. Inflammation and fibrosis are central drivers of structural injury and functional decline in DKD [42,43]. These results support the therapeutic potential of the SKR and suggest that it acts on multiple core processes in DKD progression.

Mechanistically, integrated multi-omics prioritized SLC15A2 as a key target responsive to the SKR. Although SLC15A2 is best known for peptide and drug transport [21,22], its involvement in DKD and herbal interventions has been underexplored. We found that SLC15A2 expression was reduced in DKD kidneys and selectively restored by the SKR, and this restoration was inversely associated with the accumulation of gut-derived uremic toxins [44,45,46], suggesting that enhancing proximal-tubule transport capacity may contribute to limiting nephrotoxic metabolite burden and thereby dampening downstream inflammation and fibrosis [47]. These findings extend the current understanding of SLC15A2 beyond its canonical transport functions and implicate it as a potential mediator of renoprotection in DKD.

Serum pharmacochemistry and network pharmacology identified quercetin as a core absorbed constituent of the SKR. Molecular docking, molecular dynamics simulations, and SPR consistently supported direct, high-affinity binding between quercetin and SLC15A2. In functional assays, quercetin suppressed TGF-β1-induced EMT in HK-2 cells, an effect that was abrogated by siRNA-mediated SLC15A2 knockdown [48,49,50]. These results establish SLC15A2 as a functionally necessary mediator of quercetin’s anti-fibrotic activity and collectively delineate a coherent pathway linking formula, constituent, target, and biological effect.

Nevertheless, several limitations should be acknowledged. SLC15A2 is unlikely to be the only target of the SKR, and additional pathways (for example, PI3K-Akt and AGE-RAGE signaling) may contribute to its overall efficacy. Moreover, other SKR components may act synergistically, reflecting the polypharmacology inherent to TCM formulations. Future work integrating genetic models and targeted metabolite flux analyses will help further delineate the contribution of SLC15A2 relative to other nodes in the SKR response network.

While our findings demonstrate that quercetin directly targets SLC15A2 to exert protective effects against DKD, the full spectrum of quercetin-interacting proteins remains to be elucidated. Future application of chemoproteomics strategies, such as activity-based protein profiling (ABPP) or affinity purification coupled with mass spectrometry, may enable the systematic identification of additional quercetin-binding proteins and provide a more comprehensive understanding of its multitarget mechanisms. Such approaches could also reveal previously unrecognized targets that contribute to the therapeutic efficacy of the SKR.

## 4. Materials and Methods

### 4.1. Animal Experiments

The Shen-Kang Recipe (SKR) is an empirical formula consisting of *Astragalus membranaceus*, *Rehmannia glutinosa*, *Scrophularia ningpoensis*, *Polygonatum sibiricum*, *Euryale ferox*, *Rosa laevigata*, *Smilax glabra*, *Hirudo medicinalis*, *Euonymus alatus*, *Sargassum*, and *Trachelospermum jasminoides*.

All procedures were approved by the Animal Ethics Committee of Southern Medical University (No. L2021109) and conducted in accordance with the ARRIVE guidelines and the National Institutes of Health Guide for the Care and Use of Laboratory Animals.

The Shen-Kang Recipe (SKR) is an empirical traditional Chinese medicine formula composed of eleven medicinal herbs (the complete list of constituents is provided in Table S3). Male db/db mice (8 weeks old) and their age-matched non-diabetic db/m littermates were obtained from Changzhou Cavens Model Animal Co., Ltd. (Changzhou, China). Animals were housed under specific pathogen-free conditions with a 12 h light/dark cycle and free access to standard chow and water.

After one week of acclimatization, db/db mice were randomly allocated into three groups (*n* = 6 per group): model group (receiving vehicle), low-dose SKR group (14.18 g/kg/day), and high-dose SKR group (28.36 g/kg/day). Age-matched db/m mice served as normal controls (*n* = 6). The SKR decoction or an equal volume of saline (for control groups) was administered daily by oral gavage for 12 consecutive weeks. Body weight and random blood glucose levels were monitored biweekly throughout the treatment period.

At the end of the 12-week intervention, mice were anesthetized by intraperitoneal injection of pentobarbital sodium (65 mg/kg, Sigma-Aldrich, P3761, St. Louis, MO, USA). Blood samples were collected from the orbital sinus, and serum was separated by centrifugation. Kidney tissues were rapidly excised; one portion was fixed in 4% paraformaldehyde for histological analysis, and the remaining tissue was snap-frozen in liquid nitrogen and stored at −80 °C for subsequent biochemical and omics analyses.

### 4.2. Detection of Scr and BUN Levels

Serum creatinine (Scr) and blood urea nitrogen (BUN) were measured using commercial assay kits (Creatinine Assay Kit, Jiancheng Bioengineering, C011-2-1, Nanjing, China; Urea Nitrogen Assay Kit, Jiancheng Bioengineering, C013-3-1, Nanjing, China) according to the manufacturers’ instructions. Absorbance was read at 640 nm and 546 nm using a microplate reader.

### 4.3. HE Staining

Renal tissues were harvested from the mice, fixed in 4% paraformaldehyde, embedded in paraffin, and sectioned. The obtained sections were then subjected to standard hematoxylin and eosin (H&E) staining. Subsequently, the pathological morphological changes in glomerular, tubular, and other renal structures were examined under a light microscope.

### 4.4. Masson Staining

Renal tissue sections were subjected to Masson’s trichrome staining to visualize collagen fibers in blue, allowing for the assessment of renal interstitial fibrosis. The area of blue-stained collagen fibers was quantified as a percentage of the total area using ImageJ version 1.53t (National Institutes of Health, Bethesda, MD, USA) software for quantitative analysis.

### 4.5. Immunohistochemistry

Paraffin-embedded sections underwent antigen retrieval, followed by incubation with primary antibodies against SLC15A2 (Affinity, DF14742, 1:200, Cincinnati, OH, USA), α-SMA (Proteintech, 14395-1-AP, 1:3000, Rosemont, IL, USA), KIM-1 (Proteintech, 30948-1-AP, 1:400, Rosemont, IL, USA), NGAL (Proteintech, 26991-1-AP, 1:200, Rosemont, IL, USA), N-cadherin (Proteintech, 22018-1-AP, 1:4000, Rosemont, IL, USA), and Vimentin (Proteintech, 10366-1-AP, 1:5000). Detection was performed using HRP-conjugated secondary antibodies and DAB, with hematoxylin counterstaining.

### 4.6. Proteomic Analysis

Renal tissues from db/db mice were homogenized in RIPA lysis buffer containing protease inhibitors (Roche, Basel, Switzerland). Total protein concentration was determined using a BCA assay kit (Thermo Fisher Scientific, Waltham, MA, USA). Proteins (200 μg per sample) were reduced with 10 mM dithiothreitol at 56 °C for 30 min, alkylated with 20 mM iodoacetamide in the dark for 30 min, and digested with trypsin (Promega, Madison, WI, USA) at a 1:50 enzyme-to-substrate ratio overnight at 37 °C. The resulting peptides were desalted using C18 spin columns (Thermo Fisher Scientific) and vacuum-dried.

The peptide mixtures were analyzed using a 4D label-free quantitative proteomics approach. This method integrates trapped ion mobility spectrometry (TIMS) with liquid chromatography-tandem mass spectrometry (LC-MS/MS), adding ion mobility separation as a fourth dimension to conventional retention time, *m*/*z*, and intensity dimensions. Data acquisition was performed on a timsTOF Pro mass spectrometer (Bruker Daltonics, Billerica, MA, USA) coupled with a nanoElute UHPLC system (Bruker). Peptides were separated on a 25 cm C18 column (IonOpticks, Fitzroy, Australia) with a 90 min gradient, and mass spectra were acquired in parallel accumulation-serial fragmentation (PASEF) mode.

Raw data were processed using FragPipe software (version 17.1) with the MSFragger search engine against the Mus musculus UniProt database (Swiss-Prot + TrEMBL). Search parameters included trypsin as the protease, up to two missed cleavages, carbamidomethylation of cysteine as a fixed modification, and oxidation of methionine as a variable modification. Peptide and protein identifications were filtered to a 1% false discovery rate (FDR) at both levels using the Percolator algorithm. Label-free quantification (LFQ) was performed using the IonQuant module integrated in FragPipe.

Differentially expressed proteins (DEPs) were identified based on a threshold of |log_2_FC| > 0.585 and *p*-value < 0.05 (Student’s *t*-test). The resulting DEPs were prioritized as candidate molecules for subsequent mechanistic investigation and experimental validation.

### 4.7. Wide-Target Metabolomics Analysis of Kidney Tissue

For metabolomic profiling, approximately 20 mg of kidney tissue was precisely weighed and homogenized. Metabolites were extracted using a pre-cooled methanol/acetonitrile/water mixture (2:2:1, *v*/*v*/*v*) containing 0.1% formic acid. The homogenate was vortexed, sonicated for 10 min in an ice-water bath, and centrifuged at 12,000 rpm for 15 min at 4 °C. The supernatant was collected and evaporated to dryness under a nitrogen stream, then reconstituted in 100 μL of 50% methanol for LC-MS/MS analysis. Separation was performed on an ultra-high-performance liquid chromatography (UHPLC) system (ExionLC AD, SCIEX, Framingham, MA, USA) using an ACQUITY UPLC HSS T3 column (100 mm × 2.1 mm, 1.8 μm) for hydrophobic metabolite analysis. The column temperature was maintained at 40 °C. Mobile phases consisted of water containing 0.1% formic acid (A) and acetonitrile containing 0.1% formic acid (B), with a gradient elution program: 0–2 min, 5% B; 2–15 min, 5–95% B; 15–18 min, 95% B; 18–18.1 min, 95–5% B; and 18.1–20 min, 5% B. The flow rate was 0.35 mL/min, and the injection volume was 5 μL. Mass spectrometry was performed on a QTRAP 6500+ mass spectrometer (SCIEX) equipped with an electrospray ionization (ESI) source operating in both positive and negative ion modes. Data were acquired in multiple reaction monitoring (MRM) mode. Metabolite identification was based on retention time, MS/MS fragmentation patterns, and comparison with authentic standards and the in-house mass spectral database. Raw data were processed using MultiQuant software (version 3.0.2). Differential metabolites were identified based on variable importance in projection (VIP) > 1.0 from OPLS-DA models, |log_2_FC| > 0.585, and *p* < 0.05 (Student’s *t*-test). Quality control (QC) samples were prepared by pooling equal volumes of all sample extracts and were injected periodically to monitor instrument stability; metabolites with coefficient of variation (CV) < 30% in QC samples were retained for analysis.

### 4.8. UPLC-Q-TOF/MS Analysis

The chemical profiling of SKR water extract and the identification of its absorbed prototype components in serum were performed using ultra-performance liquid chromatography coupled with quadrupole time-of-flight mass spectrometry (UPLC-Q-TOF/MS). Chromatographic separation was achieved on an ACQUITY UPLC HSS T3 column (2.1 × 100 mm, 1.8 μm) maintained at 40 °C. The mobile phase consisted of (A) 0.1% formic acid in water and (B) acetonitrile, delivered at a flow rate of 0.3 mL/min with a gradient elution program. Detection was performed on a Xevo G2-S Q-TOF mass spectrometer with an electrospray ionization (ESI) source. Data were acquired in both positive and negative ionization modes in MSE continuum mode over a mass range of *m*/*z* 50–1200. Leucine-enkephalin was used as the lock mass for real-time calibration. Components in the water extract were identified by matching accurate mass, isotopic pattern, retention time, and MS/MS fragments against commercial databases and literature. For serum samples, prototype components were confirmed by comparing their chromatographic and spectral data (precise mass, characteristic fragments, and retention time) with those in the water extract.

### 4.9. Network Pharmacology Analysis

The active ingredients of SKR and their corresponding targets were retrieved from the Traditional Chinese Medicine Systems Pharmacology Database and Analysis Platform (TCMSP, version 2.3, http://tcmspw.com/tcmsp.php). Disease targets associated with diabetic kidney disease (DKD) were retrieved from the GeneCards database (https://www.genecards.org/). The intersection between drug and disease targets was identified to construct a protein–protein interaction (PPI) network using the STRING database (https://string-db.org/). Core targets within this network were subsequently screened via topological analysis with Cytoscape software (version 3.8.1). Finally, Gene Ontology (GO) functional annotation and Kyoto Encyclopedia of Genes and Genomes (KEGG) pathway enrichment analyses for the core targets were performed using the DAVID database (https://david.ncifcrf.gov/).

### 4.10. Molecular Docking

The three-dimensional structure of quercetin, a key bioactive constituent of the SKR, was retrieved from the PubChem database (https://pubchem.ncbi.nlm.nih.gov/; CID: 5280343). The crystal structure of the core target protein SLC15A2 (peptide transporter 2) was obtained from the RCSB Protein Data Bank (PDB ID: 6VK3; resolution: 2.8 Å). Molecular docking was performed using AutoDock Vina (version 1.2.0) to predict the binding modes and binding affinity between quercetin and SLC15A2. The docking grid box was centered on the substrate-binding pocket with dimensions of 25 Å × 25 Å × 25 Å, and exhaustiveness was set to 20. The binding free energy was calculated based on the lowest-energy docking pose. The resulting protein–ligand complexes were visualized using PyMOL (version 2.5) for analysis of interaction forces, including hydrogen bonds, hydrophobic contacts, and van der Waals interactions.

### 4.11. Molecular Dynamics Simulation

To further validate the stability of the key binding conformations identified by molecular docking, molecular dynamics (MD) simulations were performed using the GROMACS 2025.2 software package. The docked ligand-receptor complex was solvated in a TIP3P water model within a periodic boundary box, and ions were added to neutralize the system. The system was neutralized by adding sodium ions. Energy minimization was performed using the steepest descent algorithm (50,000 steps) to remove steric clashes. The system was then equilibrated under NVT (constant particle number, volume, and temperature) at 300 K for 100 ps, followed by NPT (constant particle number, pressure, and temperature) at 1 bar for 100 ps, using the Parrinello-Rahman barostat. A production MD simulation was conducted for 100 ns with a time step of 2 fs, employing the AMBER ff14SB force field for the protein and GAFF2 for quercetin. The binding free energy was calculated using the Molecular Mechanics/Generalized Born Surface Area (MM/GBSA) method by extracting 100 frames from the stable trajectory (last 50 ns). This approach provides a more rigorous estimation of binding affinity than static docking scores. The simulated trajectory was analyzed for key parameters—including root mean square deviation (RMSD), root mean square fluctuation (RMSF), radius of gyration (Rg), and interaction occupancy—to comprehensively evaluate the dynamic binding stability and conformational behavior of the complex.

### 4.12. Real-Time Binding Kinetics Analysis by SPR

The real-time binding kinetics between quercetin and the SLC15A2 protein were determined by surface plasmon resonance (SPR) using a Biacore T200 system (Cytiva, Marlborough, MA, USA). The extracellular domain of recombinant human SLC15A2 protein, expressed in a mammalian system, was purified to >95% homogeneity by affinity chromatography. The purified protein was immobilized on a CM5 sensor chip (Cytiva) via amine coupling to achieve a capture level of approximately 5000 response units (RU). Quercetin (analyte) was serially diluted in running buffer (HBS-EP+, Cytiva) containing 1% DMSO to generate a five-point concentration gradient (0.156, 0.625, 2.5, 10, and 20 μM). All experiments were performed at 25 °C with a constant flow rate of 30 μL/min. Association was monitored for 60 s, and dissociation was monitored for 120 s. The sensor chip surface was regenerated using 10 mM glycine-HCl (pH 2.5) after each cycle. Data were reference- and blank-subtracted and globally fitted to a 1:1 Langmuir binding model to calculate the association rate constant (k_a_), dissociation rate constant (k_e_), and equilibrium dissociation constant (K_D_). Each experiment was independently repeated three times.

### 4.13. Cell Culture and Treatment

The human renal tubular epithelial cell line (HK-2, Procell, Wuhan, China) was routinely maintained in DMEM/F12 supplemented with 10% fetal bovine serum, 100 U/mL penicillin, and 100 μg/mL streptomycin. Cells were incubated at 37 °C in a humidified atmosphere containing 5% CO_2_. For experiments, HK-2 cells in the logarithmic growth phase and at 70–80% confluence were selected.

To investigate the protective effects of quercetin, cells were pre-treated with varying concentrations of the compound (MedChemExpress, HY-18085A, Monmouth Junction, NJ, USA) for 2 h. Subsequently, the culture medium was replaced with fresh medium containing 10 ng/mL TGF-β1 (yeasen, 91701ES10, Shanghai, China) for 48 h to induce a fibrotic phenotype, while maintaining the corresponding concentration of quercetin.

### 4.14. siRNA Transfection in HK-2 Cells

HK-2 cells were transfected with siRNA targeting human SLC15A2 (siSLC15A2; sense: 5′-CCAGAAGUCUUCAUGAAUATT-3′, antisense: 5′-UAUUCAUGAAGACUUCUGGTT-3′) or non-targeting control siRNA (siNC) using Lipofectamine 3000 according to the manufacturer’s protocol. Cells were seeded in 6-well plates and transfected at 60–70% confluence with 50 nM siRNA in Opti-MEM. After 6 h, the medium was replaced with complete growth medium. Cells were harvested 48 h post-transfection for RNA or protein extraction. Knockdown efficiency was confirmed by qRT PCR and Western blot.

### 4.15. Cell Viability Assay

Cell viability was assessed using the Cell Counting Kit-8 (CCK-8) assay. Briefly, cells were seeded into 96-well plates and subjected to the respective treatments (e.g., TGF-β1 stimulation with or without quercetin pre-treatment). Following the intervention, CCK-8 solution (NCM Biotech, C6005, Suzhou, China) was added to each well, and the plates were incubated for 0.5 to 2 h at 37 °C. The absorbance at 450 nm was then measured using a microplate reader, and the cell survival rate was calculated relative to the control group.

### 4.16. Cell Scratch Wound Healing Assay

HK-2 cells were seeded into 6-well plates and cultured until reaching over 90% confluence. A sterile pipette tip was used to create a straight scratch wound across the cell monolayer. After being gently washed with PBS to remove dislodged cells, the wells were replenished with fresh medium containing the designated treatments (e.g., TGF-β1 with or without quercetin). Images of the scratch wounds were captured at the same locations immediately (0 h) and after 24 h of incubation. The migration rate was quantified by measuring the change in the scratch area using image analysis software.

### 4.17. Immunofluorescence (IF) Staining

HK-2 cells grown on glass coverslips were fixed, permeabilized, and blocked with 5% bovine serum albumin (BSA). The cells were then incubated overnight at 4 °C with specific primary antibodies against α-SMA (Proteintech, 1:1000, 14395-1-AP) and N-cadherin (Proteintech, 1:200, 14395-1-AP). After washing, the samples were incubated with a corresponding fluorescently labeled secondary antibody (Boster, DyLight 488 Conjugated AffiniPure Goat Anti-Rabbit IgG, BA1127, Wuhan, China) at room temperature. Cell nuclei were stained with DAPI (Biosharp, BL739A, Beijing, China). Following mounting, fluorescence images were captured using an inverted fluorescence microscope to analyze the expression and localization of the target proteins.

### 4.18. Western Blotting

Total protein was extracted from cultured cells or renal tissues, and the protein concentration was determined using a bicinchoninic acid (BCA) assay. Proteins were separated by SDS-PAGE and transferred onto PVDF membranes. After blocking with 5% skimmed milk, the membranes were incubated overnight at 4 °C with the following primary antibodies: anti-SLC15A2 (Affinity, 1:1000, DF14742), anti-E-cadherin (Proteintech, 1:5000, 20874-1-AP), anti-α-SMA (Proteintech, 1:6000, 14395-1-AP), anti-N-cadherin (Proteintech, 1:5000, 22018-1-AP), anti-Vimentin (Proteintech, 1:10,000, 10366-1-AP) and anti-β-actin (Proteintech, 1:5000, 20536-1-AP). Subsequently, the membranes were incubated with HRP-conjugated secondary antibodies (Boster, BM2006/BA1050) at room temperature. Protein bands were visualized using an ECL chemiluminescence kit and imaged with a chemiluminescence detection system. The grayscale values of the bands were quantified using ImageJ software.

### 4.19. Quantitative Real-Time PCR (qRT-PCR)

Total RNA was extracted from cells or renal tissues using AG RNAex Pro Reagent (AG Bio, AG21101, Changsha, China) and subsequently reverse-transcribed into complementary DNA (cDNA) using a PrimeScript RT reagent Kit (AG Bio, AG11711). The qRT-PCR amplification was performed using a SYBR Green premix reagent (AG Bio, AG11701) on a real-time PCR detection system. The relative mRNA expression levels of the target genes were normalized to β-actin as an internal control and calculated using the 2^−ΔΔCt^ method. Primers were designed according to the National Center for Biological Information (NCBI) Primer-BLAST, https://www.ncbi.nlm.nih.gov/tools/primer-blast/ (see Table S4 for sequences) and synthesized by Biotech Bioengineering Co. (Shanghai, China).

### 4.20. Statistical Analysis

All experiments were repeated at least three times independently. Data are presented as the mean ± SD. Statistical analyses were performed using GraphPad Prism software (version 9.0). Comparisons between two groups were analyzed using an unpaired Student’s *t*-test, while comparisons among multiple groups were conducted by one-way analysis of variance (ANOVA) followed by an appropriate post hoc test. A *p*-value < 0.05 was considered significant.

## 5. Conclusions

In summary, this study demonstrates that the Shen-Kang Recipe (SKR) attenuates renal fibrosis and improves renal function in diabetic kidney disease by modulating the peptide transporter SLC15A2. Integrated multi-omics analysis identified SLC15A2 as a key proximal tubule protein downregulated in DKD and selectively restored by the SKR. Quercetin, a major absorbed constituent of the SKR, was shown to directly bind to SLC15A2 with high affinity and to exert anti-fibrotic effects in an SLC15A2-dependent manner. These findings establish SLC15A2 as a functionally necessary mediator of quercetin’s anti-fibrotic activity and provide a mechanistic basis for the therapeutic efficacy of SKR. Future chemoproteomics approaches may further elucidate the full spectrum of quercetin-interacting proteins and expand the understanding of its multi-target mechanisms in DKD.

## Data Availability

The proteomics and metabolomics raw data generated in this study have been deposited in the OMIX database, China National Center for Bioinformation/Beijing Institute of Genomics, Chinese Academy of Sciences (https://ngdc.cncb.ac.cn/omix, accessed on 16 March 2026), under the accession number OMIX015618 (Proteomics) and OMIX015619 (Metabolomics). All other data supporting the findings of this study are available within the article and its Supplementary Materials. Additional data are available from the corresponding authors upon reasonable request.

## Supplementary Materials

The following supporting information can be downloaded at https://www.mdpi.com/article/10.3390/ijms27073291/s1.

## Abbreviations

The following abbreviations are used in this manuscript:

DKD	Diabetic kidney disease;
ESRD	End-stage renal disease;
SKR	Shen-kang Recipe;
TGF-β1	Transforming growth factor-β1;
TCM	Traditional Chinese Medicine;
EMT	Epithelial-mesenchymal transition;
KIM-1	Kidney injury molecule-1;
NgaL	Neutrophil gelatinase-associated lipocalin;
a-SMA	α-Smooth Muscle Actin;
TPP1	Tripeptidyl peptidase I;
Ang II	angiotensin II;
H&E	Hematoxylin and eosin;
IHC	Immunohistochemistry;
DAB	Diaminobenzidine;
TCMSP	Traditional Chinese Medicine Systems Pharmacology Database and Analysis Platform;
CCK-8	Cell Counting Kit-8;
IF	Immunofluorescence;
PAGln	Phenylacetyl-L-Glutamine;
TMAO	Trimethylamine-N-Oxide;
UPLC-Q-TOF/MS	Ultra-high performance liquid chromatography-quadrupole time-of-flight mass spectrometry.
