# Supplementary material for: Multi-Omics and Functional Validation Identify a Quercetin-SLC15A2 Axis That Mediates the Anti-Fibrotic Effect of Shen-Kang Recipe in Diabetic Kidney Disease
Source: Int J Mol Sci. 2026 Apr 5;27(7):3291. doi: 10.3390/ijms27073291 (PMC13072949; doi:10.3390/ijms27073291)
Supplement: Supplementary file 1 [file ijms-27-03291-s001.zip › ijms-4198677-supplementary.pdf]

Supplementary Material

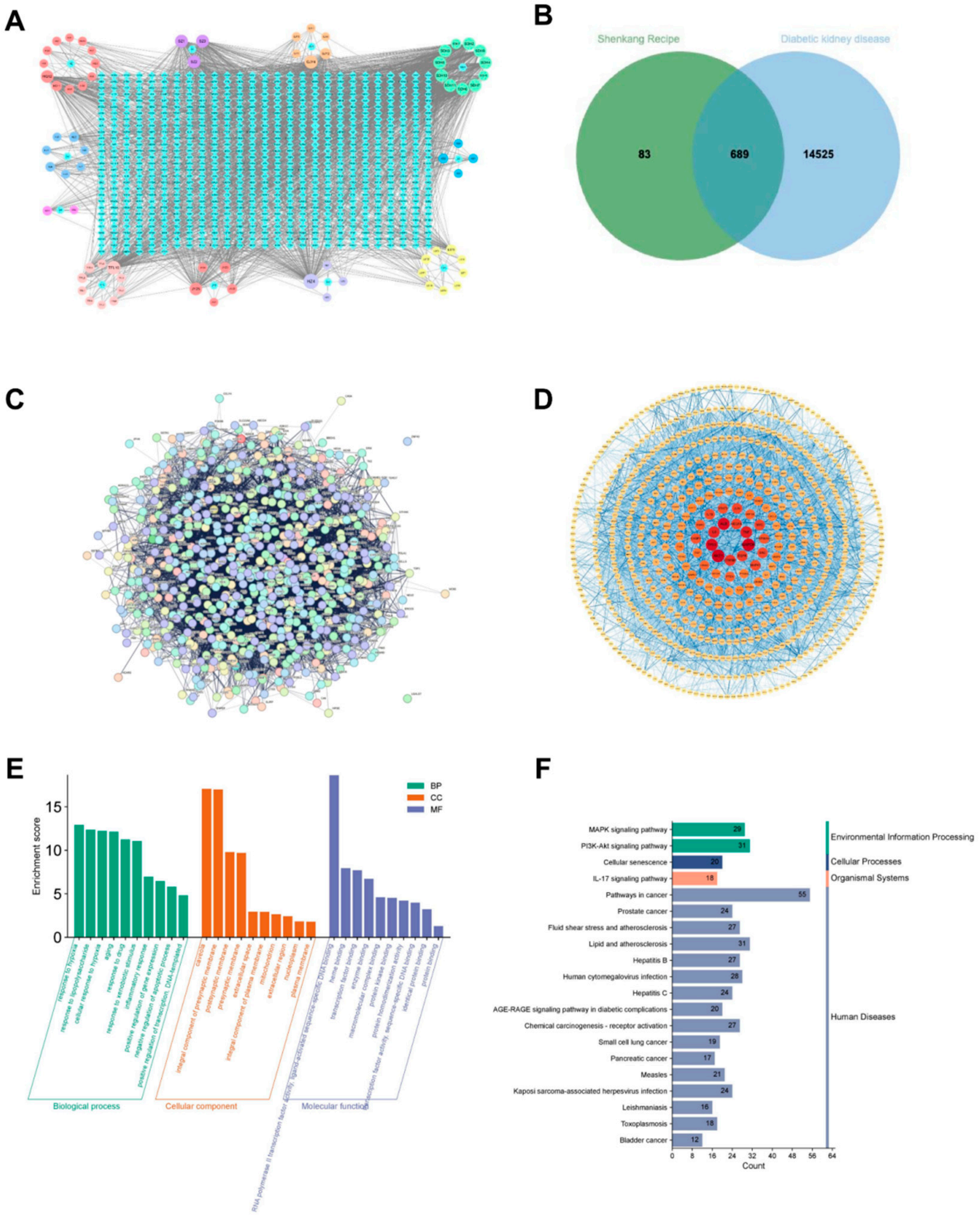

Figure S1. Network pharmacology analysis identifies key active components and targets of SKR against DKD.(A) Active component-target network diagram. (B) Venn diagram illustrating the overlap between SKR potential targets and known DKD-related targets. (C) Active component-disease protein-protein interaction (PPI) network. (D) Intersecting target protein interaction network diagram. (E) Gene Ontology (GO) enrichment analysis (top 10). (F) KEGG pathway enrichment analysis (top 20).

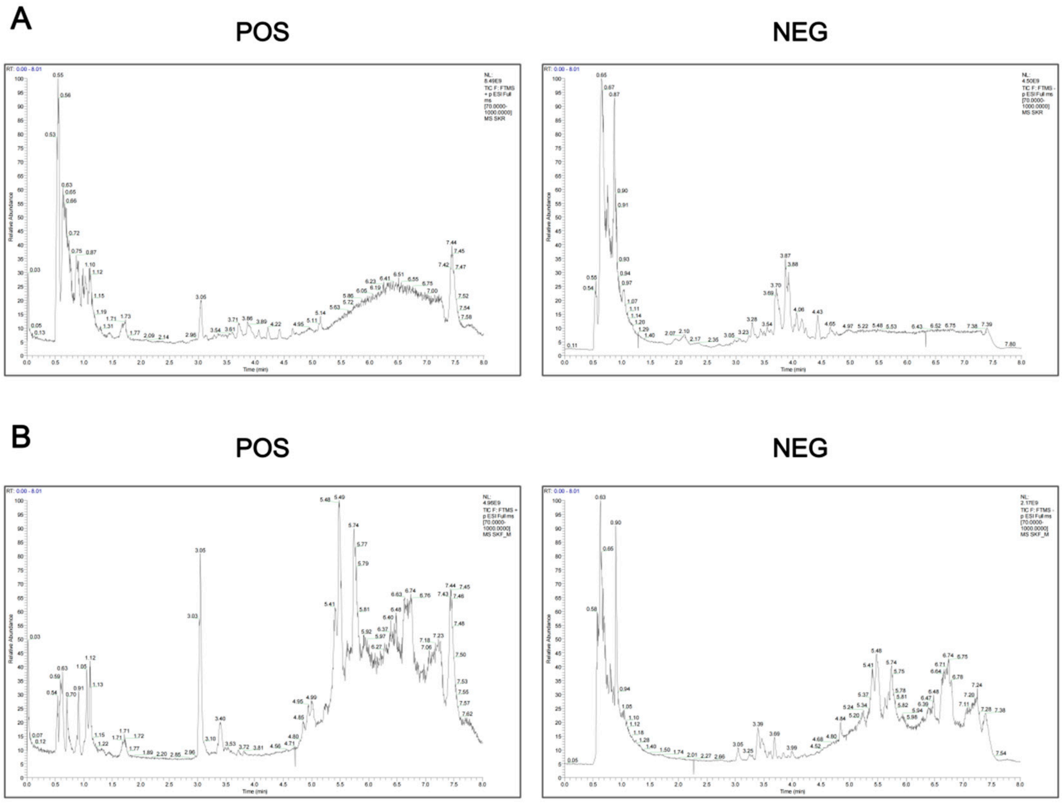

Figure S2. Identification of Quercetin as a prototype component of SKR in serum via UPLC-Q-TOF/MS analysis. (A) Total Ion Chromatogram (TIC) of the water extract of Shen-Kang Recipe. Based on comparisons with mass spectrometry databases, 48 major constituents were preliminarily identified, including flavonoids (e.g., Quercetin, Dihydrokaempferol, and Astilbin), organic acids, and saponins. (B) Identification of prototype components from SKR detected in mouse serum after administration. Quercetin was consistently detected with relatively high signal intensity, indicating its role as a critical directly compound in vivo.

Table S1. Key active components in SKR based on degree value

Rank	Degree	CAS Number	Compound Name
1	153	117-39-5	Quercetin
2	114	83-48-7	Stigmasterol
3	104	58-96-8	Uridine
4-14	101	24512-63-8	Methyl

		(4aR,7aS)-7-(hydroxymethyl)-1-[3,4,5-trihydroxy-6-(hydroxymethyl)oxan-2-yl]oxy-1,4a,5,7a-tetrahydrocyclopenta[c]pyran-4-carboxylate
101	79638-01-0	Genipinic acid
101	25019-41-4	Methyl (2E,8Z)-deca-2,8-dien-4,6-diynoate
101	118-00-3	Guanosine
101	1378978-74-5	Diincarvilone B
101	103744-81-6	Rehmaglutin C
101	141040-72-4	3-Hydroxy-2,6,6-trimethylcyclohexene-1-carboxylic acid
101	103744-83-8	Rehmaglutin B
101	531-29-3	Coniferin
101	10338-51-9	Salidroside
101	771-50-6	Indole-3-carboxylic acid

Components are ranked by degree value. Ranks 4 to 14 share an identical degree value of 101 and are thus considered equally important based on this metric.

Table S2. Hydrogen bond analysis of the quercetin–SLC15A2 complex from molecular dynamics simulations

Acceptor	DonorH	Donor	Frames	Frac	AvgDist	AvgAng
MOL_457@O27	TRP_405@HE1	TRP_405@NE1	7869	0.7869	3.0282	154.9239
MOL_457@O28	TYR_19@HH	TYR_19@OH	6143	0.6143	2.8638	55.8618

Table S3. Composition of Shen-Kang Recipe (SKR)

Chinese name	Ingredients	Medicinal parts	Amount(g)
Huang-Qi	*Astragalus menbranaceus (Fisch) Bge. var. Mongholicus (Bge.) Hsiao*	The dried root of *Astragalus menbranaceus.*	60
Sheng-Di-Huang	*Rehmannia glutinosa Libosch*	The dried root tuber of *Rehmannia glutinosa*	15
Xuan-Shen	*Scrophularia ningpoensis Hemsl.*	The root of *Scrophularia ningpoensis*	15
Shui-Zhi	*Hirudo nipponica Whitman*	The dried total body of *Hirudo nipponica*	10
Gui-Jian-Yu	*Euonymus alatus (Thunb.) Sieb*	The dried winged branch of *Euonymus alatus*	15
Huang-Jing	*Polygonatum sibiricum Red.*	The dried rootstock of *Polygonatum sibiricum*	15
Hai-Zao	*Sargassum pallidum(Turn.) C.Ag.*	The dried algal body of *Sargassum pallidum*	15
Tu-Fu-Ling	*Smilax glabra Roxb.*	The dried rootstock of *Smilax glabra*	15
Qian-Shi	*Euryale ferox Salisb.*	The dried ripe seed kernel of *Euryale ferox*	15
Jin-Ying-Zi	*Rosa laevigata Michx.*	The dried ripe fruit of *Rosa laevigata*	15
Luo-Shi-Teng	*Trachelospermum jasminoides(Lindi.)Le*	The Dried leafy rootstock of *Trachelospermum*	30

m. *jasminoides*

Table S4. Primer sequences for qRT-PCR

**Gene name**	**Primer sequences**
KIM-1 (mouse)	F:ATGGCAAAGTCTTTCCGTGT
	R:TCCACACAGCCACCAGTATC
NgaL (mouse)	F:TGGCCCTGAGTGTCATGTG
	R:CTCTTGTAGCTCATAGATGGTGC
IL-6 (mouse)	F:TAGTCCTTCCTACCCCAATTTCC
	R:TTGGTCCTTAGCCACTCCTTC
IL-1β (mouse)	F:GCAACTGTTCCTGAACTCAACT
	R:ATCTTTTGGGGTCCGTCAACT
TNF-α (mouse)	F:CCCTCACACTCAGATCATCTTCT
	R:GCTACGACGTGGGCTACAG
a-SMA (mouse)	F:GTCCCAGACATCAGGGAGTAA
	R:TCGGATACTTCAGCGTCAGGA
N-cadherin (mouse)	F:TGCCATTAGCCAAGGGAATAG
	R:GCGTTCCTGTTCCACTCAT
SLC15A2 (mouse)	F:TCATCGTGGCTGTGCTCATC
	R:CAGGTAGCCAAACACGATGC
β-actin (mouse)	F:GGCTGTATTCCCCTCCATCG
	R:CCAGTTGGTAACAATGCCATGT
E-cadherin (Human)	F:CGAGAGCTACACGTTCACGG
	R:GGGTGTCGAGGGAAAAATAGG
a-SMA (Human)	F:CAGGGCTGTTTTCCCATCCAT

	R:GCCATGTTCTATCGGGTACTTC
Vimentin (Human)	F:AGTCCACTGAGTACCGGAGAC
	R:CATTTCACGCATCTGGCGTTC
N-cadherin (Human)	F:CCTCCAGAGTTTACTGCCATGAC
	R:GTAGGATCTCCGCCACTGATTC
SLC15A2 (Human)	F:GCTGCTGGTGATCCTCATCT
	R:AGGATGCCACACTTGACCAT
β-actin (Human)	F:CATGTACGTTGCTATCCAGGC
	R:CTCCTTAATGTCACGCACGAT
